# Evolutionary Expansion of WRKY Gene Family in Banana and Its Expression Profile during the Infection of Root Lesion Nematode, *Pratylenchus coffeae*

**DOI:** 10.1371/journal.pone.0162013

**Published:** 2016-09-07

**Authors:** Raja Kaliyappan, Sriram Viswanathan, Backiyarani Suthanthiram, Uma Subbaraya, Saraswathi Marimuthu Somasundram, Mayilvaganan Muthu

**Affiliations:** Crop Improvement Division, ICAR-National Research Centre for Banana, Tamil Nadu, Tiruchirapalli, India; Bhabha Atomic Research Centre, INDIA

## Abstract

The WRKY family of transcription factors orchestrate the reprogrammed expression of the complex network of defense genes at various biotic and abiotic stresses. Within the last 96 million years, three rounds of *Musa* polyploidization events had occurred from selective pressure causing duplication of *MusaWRKYs* with new activities. Here, we identified a total of 153 WRKY transcription factors available from the DH Pahang genome. Based on their phylogenetic relationship, the MusaWRKYs available with complete gene sequence were classified into the seven common WRKY sub-groups. Synteny analyses data revealed paralogous relationships, with 17 *MusaWRKY* gene pairs originating from the duplication events that had occurred within the *Musa* lineage. We also found 15 other *MusaWRKY* gene pairs originating from much older duplication events that had occurred along Arecales and Poales lineage of commelinids. Based on the synonymous and nonsynonymous substitution rates, the fate of duplicated *MusaWRKY* genes was predicted to have undergone sub-functionalization in which the duplicated gene copies retain a subset of the ancestral gene function. Also, to understand the regulatory roles of *MusaWRKY* during a biotic stress, Illumina sequencing was performed on resistant and susceptible cultivars during the infection of root lesion nematode, *Pratylenchus coffeae*. The differential *WRKY* gene expression analysis in nematode resistant and susceptible cultivars during challenged and unchallenged conditions had distinguished: 1) *MusaWRKY*s participating in general banana defense mechanism against *P*.*coffeae* common to both susceptible and resistant cultivars, 2) *MusaWRKY*s that may aid in the pathogen survival as suppressors of plant triggered immunity, 3) *MusaWRKY*s that may aid in the host defense as activators of plant triggered immunity and 4) cultivar specific *MusaWRKY* regulation. Mainly, *MusaWRKY*52, -69 and -92 are found to be *P*.*coffeae* specific and can act as activators or repressors in a defense pathway. Overall, this preliminary study in *Musa* provides the basis for understanding the evolution and regulatory mechanism of *MusaWRKY* during nematode stress.

## Introduction

Dynamic cellular reprogramming is a plant defense response to pathogen infection [1]. The WRKY family of proteins function as transcriptional regulator and orchestrate the reprogrammed expression of the labyrinthine network of defense genes as an induced defense response. Also, WRKY transcription factors function in regulating multiple processes ranging from seed germination to secondary metabolism [2, 3]. In general, WRKY transcription factors control the gene expression by binding to the TTGAC(C/T) W-box cis-element in the promoter region of target genes and function as activators or repressors. Several members of WRKY transcription factors had been shown to modulate expression of genes involving in pathogen-associated molecular pattern (PAMP) triggered immunity [4, 5] and effector-triggered immunity [6, 7]. High-affinity DNA binding takes place via their highly conserved amino acid sequence WRKYGQK at the N-terminus and the zinc finger-like motif Cys(2)-His(2) or Cys(2)-HisCys at the C-terminus, which are the characteristic features of WRKY family of proteins. WRKYs may also contain HARF, LZ, LXXL, LXLXLX motifs [8] and proline-directed serine (SP) clusters making them suitable for modifications like phosphorylation [9], acetylation [10] and dimer formation [11]. Identifying and manipulating of this master transcriptional factor during a pathogen attack in banana, the only crop grown in most number of countries [12] with a huge economic importance especially for developing countries, would provide a foundation for deciphering the role of WRKYs in *Musa* defense mechanisms. *Musa* has the highest number of transcription factors among all sequenced plant genomes with a tremendous expansion of WRKY gene family during evolution. *Musa* contains the second largest WRKY family (153 members) next to *Glycine max* (176 members) [13]. These numbers signify the need for WRKY transcription factors by *Musa* for a fittest survival amidst various biotic and abiotic factors.

*Pratylenchus coffeae* is a major destructive nematode of banana [14] that is widespread in tropical regions where 78% of bananas were grown [12]. *P*. *coffeae* penetrate and migrate through root causing extensive necrosis of root cortical parenchyma and endodermal cells by direct feeding and this root damage also leads to an avalanche of infections via invasion of other phytopathogenic microorganisms. Intense *P*. *coffeae* root damages hinder the absorption of water and nutrients from the soil causing stunting, chlorosis in leaves, even toppling of the plant due to weak anchorage by extensively damaged root system and significant yield loss in banana production [14]. *P*. *coffeae* is an obligate parasite and compulsorily needs to exploit a host for its reproduction to complete the lifecycle. These nematodes feed on the contents of host cells without the luxury of forming specialized feeding structures like synctia, cysts and giant cells as formed by other root knot nematodes and cyst nematodes [14, 15]. Such traits are the reflection of its remarkably smaller genome sized at only ~20Mb, the smallest known animal genome ever. However, with high gene density these obligatory nematodes originating >600 million years ago (MYA) have evolved well for their existence in banana plants that originated around 75 MYA. Interestingly, plant parasitism has spiked up at least four times independently within the nematode clade Tylenchida comprising *P*. *coffeae* [16]. For example, *P*. *coffeae* uses stomatostylet, a unique stylet type of the suborder Tylenchina, to puncture holes for entering into cells and ingests cytoplasm directly. For modifying and loosening plant cell walls, an array of cell wall-degrading enzymes like cellulases, xylanases, polygalacturonases, β,1,3- endoglucanase, pectate lyases and expansins are putatively secreted by the subventral gland cells of nematodes to assist the stylet insertion [17–19]. As a counter attack, nematode secreted effector proteins/peptides are recognized by the root cells followed by initiation of defense signaling cascade and regulation of defense-related genes. Adaptive responses of *Musa* to *P*.*coffeae* include triggering lignin production, ROS burst and induced defense signaling events like ethylene response [14]. WRKYs are well known for the activation and suppression of such defense signaling mechanisms. Many WRKYs had been reported to show resistance against various nematodes in rice, arabidopsis, tomato and soybean (S1 Table) and known for triggering a multitude of defense response genes. To gain an insight into nematode-*Musa* interactions, genome-wide expression pattern of *MusaWRKY* in both nematode resistant and susceptible cultivars were analyzed during the infection of a mixed life stage population of *P*. *coffeae*, by performing Illumina sequencing. The expression analysis of *MusaWRKY* genes showed that *MusaWRKYs* are involved in the nematode stress response. Along with the study on evolution and duplication of *MusaWRKY* genes, this paper provides a foundation for further functional studies on *WRKY* genes in banana.

## Materials and Methods

### MusaWRKYs and Phylogenetic analysis

The 153 *Musa acuminata* WRKY protein and nucleotide sequences were obtained from the Banana Genome Hub (http://banana-genome.cirad.fr/). *Arabidopsis* WRKY protein sequences were obtained as reference from The Arabidopsis Information Resource (TAIR). Complete CD sequences were separated from the partial fragment sequences available at the Banana Genome Hub. The 60 amino acid long complete WRKY and Zinc finger domains of 81 MusaWRKY proteins were used to create multiple protein sequence alignments using ClustalW. The neighbor-joining method was used to construct the phylogenetic tree based on amino acid sequence of WRKY domains using MEGA 5.2.1 software (Bootstrap value 1000). Analysis for conserved motifs in the WRKY proteins was carried out using MEME database (http://meme.sdsc.edu/meme/cgi-bin/meme.cgi). It was observed that most conserved domains are limited to a single subfamily of WRKY transcription factors and therefore MEME analyses were run for the members of each subfamily using the protein sequences (S11 Table). The online program 2ZIP (http://2zip.molgen.mpg.de/index.html) was used for predicting Leu zipper motifs, while HARF, LXXLL and LXLXLX motifs were identified by manual inspection.

### Chromosomal location of MusaWRKYs and evolution

All *MusaWRKY* genes were mapped to *Musa* chromosomes based on information available at The Banana Genome Hub (http://banana-genome.cirad.fr/). Tandem clusters located within 50kb on individual chromosomes were identified from the gene positions on the scaffolds taken from the plant genome duplication database (http://chibba.agtec.uga.edu/duplication/index/files). To determine paralogous *WRKY* gene pairs between *Musa* chromosomes that resulted from the last three rounds of whole genome duplication (WGD), we used “Syntenic DotPlot” section of The Banana Genome Hub (http://banana-genome.cirad.fr/dotplot). To visualize the synteny, we did all chromosomes by all chromosomes analysis and obtained the dot plot. The list of co-localized paralogs with synonymous and nonsynonymous substitution rate values were obtained and the paralogous WRKY gene pairs were linked on the WRKY karyotype locus map obtained from the locus search on The Banana Genome Hub.

### Nematode culture

Nematode infected banana roots were collected from the research farm at the ICAR-National Research Centre for Banana, India. Roots were chopped and incubated in water. Then nematodes were extracted through sieves [14] and examined under light microscope (Olympus SD-STB3, Japan). Based on morphological appearance 100 number of female *P*. *coffeae* alone were picked out and inoculated into carrot discs on 1% agar medium. It was maintained at 26°C and after 45 days, nematodes were collected and used for inoculation in the banana roots.

### Plant materials and sample collection

Uniformed sized suckers of banana resistant cultivar, Karthobiumthum (ABB) (NRCB-0050) and susceptible cultivar, Nendran (AAB) (NRCB-0615) were collected and treated with nematicide. These suckers were planted in the cement pots containing fumigated pot mixture in the ratio of 1:1:1 (sand:farmyard manure: red soil) and maintained in the glass house. One month after planting, individual plastic cup containing hole on the side was placed by removing the soil near the plants in pots and those cups were filled with potting mixture. Single root was selected in each plant and carefully inserted through the hole into the plastic cup. Each cup was inoculated with 3000 active root lesion nematodes. Inoculated roots were collected at various time points (2, 4, 6, 8 and 10 days post inoculation) in three replications for each time interval from independent plants of both resistant and susceptible cultivars. Similarly, the root samples were collected at the same time points from uninocluated plants. Collected roots were washed with DEPC water and frozen by using liquid nitrogen and kept in -80°C for later analysis.

### Construction of cDNA library and Illumina deep sequencing

Total RNA was extracted from each sample (2 gram) using Agilent Plant RNA isolation mini kit (Agilent Technologies, Inc., USA) (product no. 5188–2780). The RNA was treated with DNase I and their integrity was tested. Equal amount of RNA corresponding to root samples collected at various time intervals (2, 4, 6, 8 and 10 days) were pooled together separately for resistant and susceptible cultivars from both inoculated and uninoculated plants. This pooled RNA samples of nematode inoculated and uninoculated resistant and susceptible cultivars were used for next generation sequencing and quantitative real time PCR validation (qRT-PCR). The library construction and sequencing were performed by the Genotypic Technology, Bangalore, India, according to the Illumina TruSeq RNA library protocol outlined in ‘‘TruSeq RNA Sample Preparation Guide” (Part # 15008136; Rev. A; Nov 2010). The prepared library was quantified using Nanodrop and validated for quality by running an aliquot on High Sensitivity Bioanalyzer Chip (Agilent). The library was loaded onto the channels of an Illumina HiSeqTM GAII Analyzer instrument for 4 gigabase in-depth sequencing, which was used to obtain more detailed information about gene expression. Each paired-end library had an insert size of 200–700 bp. The average read length of 90 bp was generated as raw data. Raw sequence data were generated by Illumina pipeline and were available in NCBI’s Short Read Archive (SRA) database (http://www.ncbi.nlm.nih.gov/sra/SRP071591) under accession number SRP071591. Information about the sequencing data like depth and number of contigs is listed in the S1 Table.

### Genome assembly, sequence clustering and digital gene expression (DGE)

The clean reads were obtained from raw data by filtering out adaptor-only reads from reads containing more than 5% unknown nucleotides (nt), and low-quality reads. Then, clean reads were mapped to the reference AA genome (*Musa acuminata*) with TopHat (v2.0.4) [20] and their relative abundance were calculated using cufflinks (v2.0.1) and then annotated independently. Annotated results of WRKY genes were further shortlisted based on the reference genome ID. The differential expression of the genes among challenged and unchallenged libraries of resistant and susceptible cultivars was calculated based on fold value which was obtained from their respective FPKM values. The summation of FPKM values for every transcript associated with a particular gene gives the expression measurement in FPKM. The differential gene expression is calculated by the Cuffdiff program using the ratio of uninoculated vs. incoculated FPKM values for every gene. The following cutoff was assigned for upregulated and downregulated genes based on the P value < 0.05 (FC is log fold change to the base 2).

### Quantitative Real Time PCR (qRT-PCR)

For qRT-PCR analysis, 1 μg of RNA was used for cDNA synthesis by first strand reverse transcription kit with oligo-(dT) primers according to manufacturer’s protocol (Promega). The first strand cDNA was diluted (1:10) with water and was used as template for qRT-PCR. Six MusaWRKYs (69, -52, -92, -41, -19 and -81) were selected for validation. Specific gene primers (S2 Table) were designed using PRIMER3 software. Three independent PCR reactions were carried out for each gene. The PCR reactions were performed in a 20 μL total reaction volume including 10 μL of Power SYBR Green PCR Master Mix (Applied Biosystem), 5 mM each of gene-specific primers, and 1 μL of cDNA templates. The detection was carried out by LightCycler 480 (Roche, Germany) system and the following programme was used: 10 min/94°C; then 40 cycles of 20 s/94°C, 30 s/56°C, and 20 s/72°C; and finally for melting curve analysis: 1 min/95°C, 30 s/56°C, and 30 s/95°C was used. The relative level of each target gene was normalized by comparing the copy numbers of target mRNA with RPS2. The relative expression level was calculated using the 2^-ΔΔCt^ method.

## Results and Discussion

### *MusaWRKY* family and phylogeny

**Fig 1:**
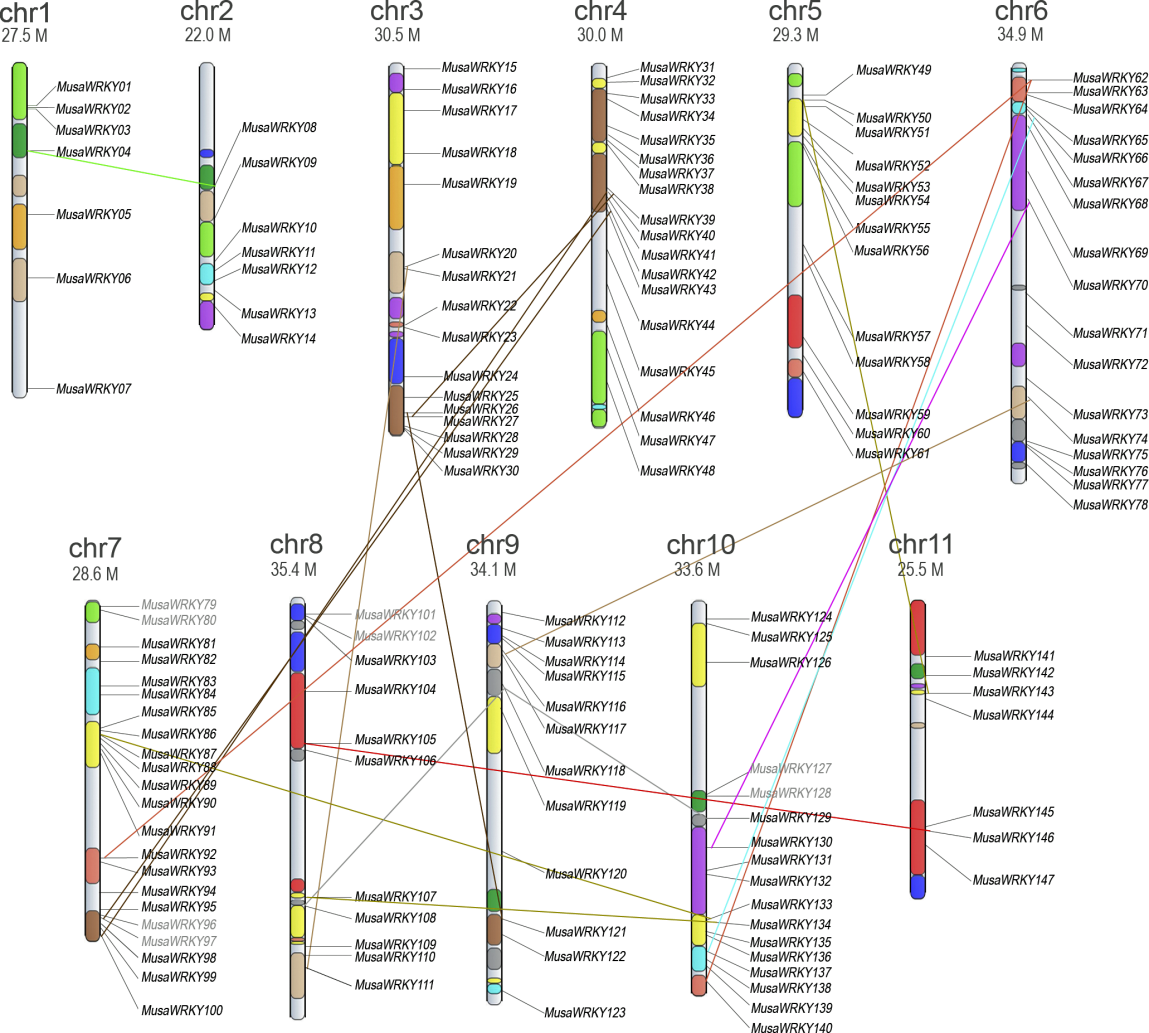
Chromosomal distribution of WRKY genes in Musa. Eleven synteny blocks of *Musa* chromosomes are represented as in the banana genome hub.The paralogous chromosome segments are represented in the same colors. The 17 paralogs are linked by the same colored lines. Number and size of the each chromosome are given at the top.

**Fig 2:**
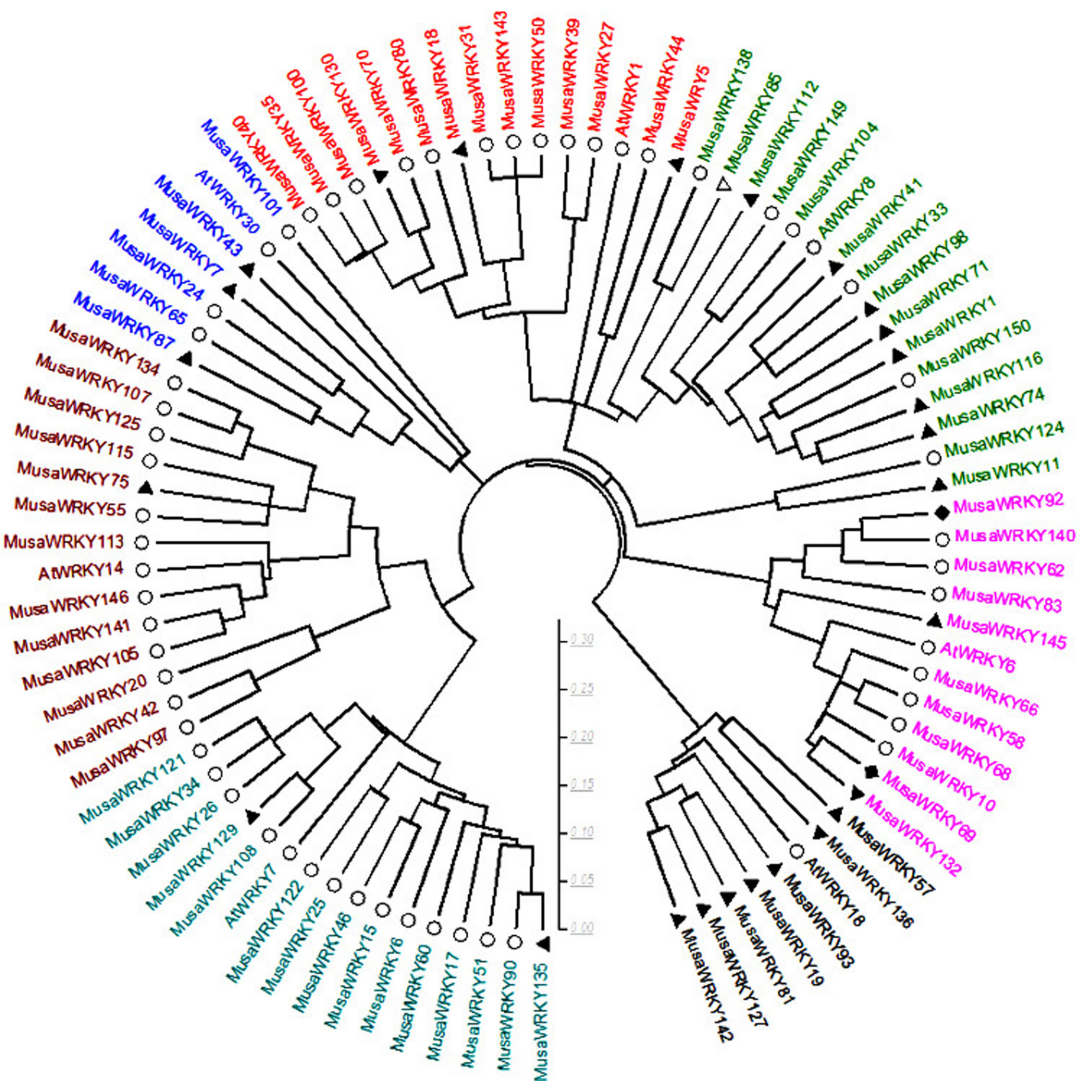
Unrooted phylogenetic tree representing relationships among WRKY domains of *Musa* and *Arabidopsis*. Amino acid sequences of WRKY genes were aligned with clustal W and 1000 bootstrap values in neighbor-joining method in MEGA 5.2.Branch name of sub trees are colored indicating different WRKY groups and subgroups. Triangle symbols indicated that significant expression in susceptible or resistant cultivars. Ο symbol shows that no genes were expressed, and arcs symbol showed that up-regulated in resistant and down-regulated in susceptible cultivar during nematode infection.

**Fig 3:**
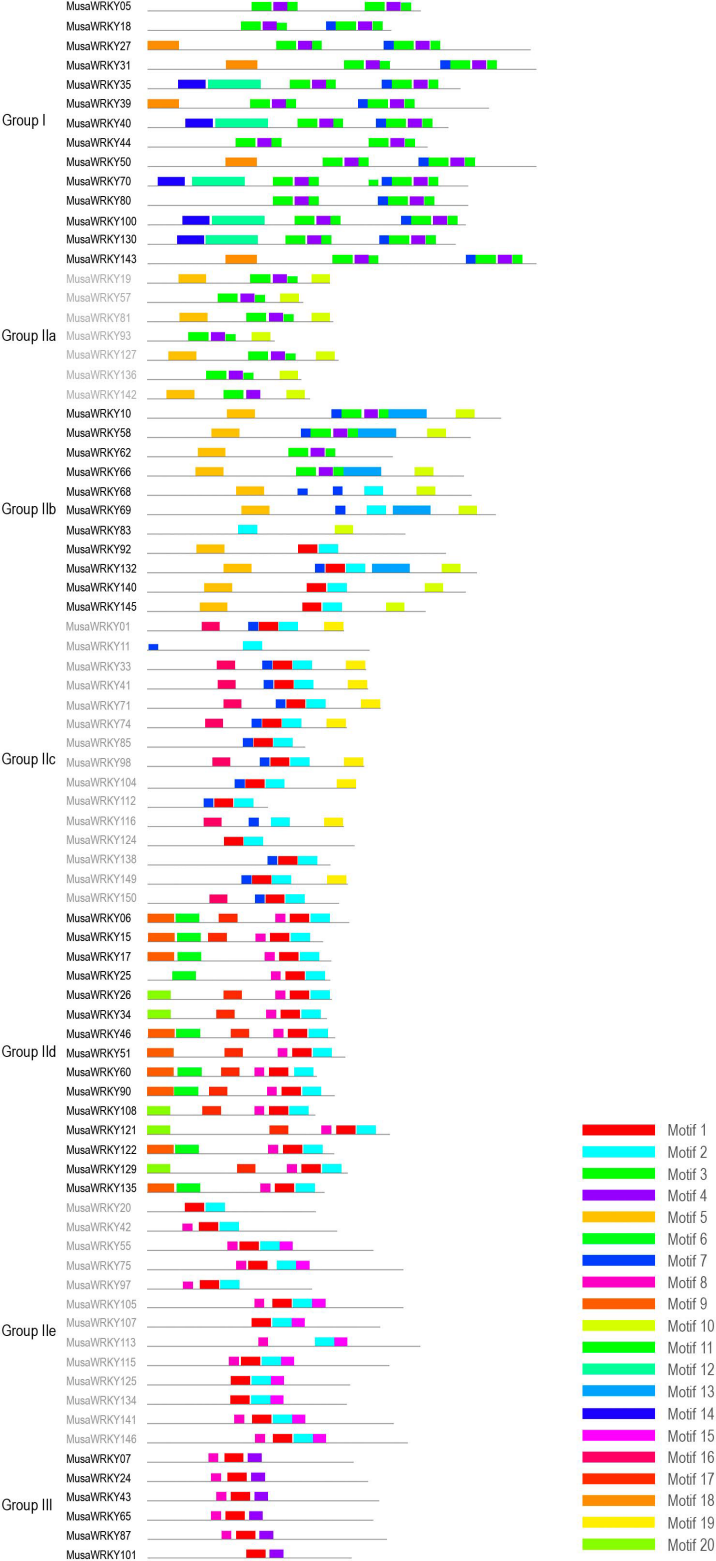
Conserved motifs of 81 Musa WRKY proteins. MEME was used to predict motifs and these motifs represented with boxes.

A total of 153 *WRKY* sequences were retrieved from the Banana Genome Hub (CIRAD, France), of which only 81 *WRKY*s had complete CDs. *WRKY* members were dispersed on 11 *Musa* chromosomes (Fig 1). Numbering of many published *MusaWRKYs* correspond to its orthologous *Arabidopsis* gene and may result in a *WRKY* to have two or more different names. So, we designated 153 *WRKY*s as *MusaWRKY1* through *MusaWRKY147* based on their orders on chromosomes and six *MusaWRKY* genes that may be located on unanchored scaffolds and could not be mapped to any chromosomes were designated as *MusaWRKY148* through *MusaWRKY153*(S3 Table, Fig 1). To avoid false positives, only the 81 MusaWRKYs with complete CDs were classified into three major groups (Table 1, Fig 2) based on the number of WRKY domains (single or double), additional amino acid motifs and phylogeny [21]. Accordingly, all group I members had two heptapeptide WRKY domains (WRKYGQK). The presence of SP clusters were also noticed among them which is a characteristic feature of group I WRKYs [22].Group I MusaWRKYs comprised 17% of the total Musa*WRKY* family and were comparable to group I WRKYs of *Brachypodium distachyon* (19%) and *Oryza sativa* (15%) belonging to commelinids clade of monocots [23, 24]. Based on the amino acid motifs outside the WRKY domain, Group II MusaWRKYs were further divided into 5 subgroups: Group IIa, IIb, IIc, IId and IIe. Unique motif and shared motif composition was observed among MusaWRKY members within the same subgroups (Fig 3, S4 Table). Group IIa is considered to be the recently evolved WRKY group [25] and in *Musa*, 7 members were present in this group. Noticeably, motifs 5 and 10 were limited to the members of group IIa and group IIb, whereas only group IIc members contained motifs 16 and 19. Group IIc had undergone a significant expansion containing more than 50% of the Musa*WRKY* family. The HARF motif was present only among the members of group IId. Motif 15 existed uniquely in group II-e. Group I and group II WRKYs shared the same C2-H2 type zinc finger motif (C–X_4–7_–C–X_23–29_–H–X_1_–H). In general, group III *WRKY* genes were believed to have specific roles in monocotyledonous plants [24] and in *Musa*, 6 WRKYs were present in this group with C2-HC-type zinc finger motif (C–X_4–7_–C–X_23–29_–H–X_1_–C). In group III, MusaWRKY43 showed divergence in the WRKY domain and had WRKYGYK. A variety of domains like LXXL (MusaWRKY18, -5, -2, -7, -80, -129, -75, -24 and -87), LZ (MusaWRKY10, -68, and -145), HARF (MusaWRKY135, -122, -90, -60, -46, -25, -17, -15 and -6) and LXLXLX (MusaWRKY6, -68, -81, -127, -62, -92, -140, -51 and -43) were present (S4 Table) and MusaWRKY family of proteins displayed fundamental physiochemical properties with wide ranges (S5 Table) together supporting that different MusaWRKYs can interact with different substrates to perform a wide variety of cellular functions. For example, the interaction of WRKY with MAPKs occurs at the SP (Serine-Proline) clusters located next to a D-domain, which is a MAPK docking motif. This interaction had been proved for effective phosphorylation of WRKY in plants and MusaWRKY18 belonging to group I have SP clusters and D-domain that can be phosphorylated by the MAPK for the activation of defense-related genes [26, 27].

**Table 1:** WRKY TF's identified in Musa with complete CD's.

*WRKY* Name	Chr	*Musa* Genome Hub ID	Conserved motif	Group	Domain Pattern	Domains	SSR Motif
*MusaWRKY5*	1	GSMUA_Achr1T17160_001	WRKYGQK	Group I	C-X4-C-X22-H-X1-H	LXXL	-
*MusaWRKY18*	3	GSMUA_Achr3T09940_001	WRKYGQK	Group I	C-X4-C-X22-H-X1-H	LXXL	1
*MusaWRKY27*	3	GSMUA_Achr3T29850_001	WRKYGQK	Group I	C-X4-C-X22-H-X1-H	-	-
*MusaWRKY31*	4	GSMUA_Achr4T01600_001	WRKYGQK	Group I	C-X4-C-X22-H-X1-H	-	-
*MusaWRKY35*	4	GSMUA_Achr4T07230_001	WRKYGQK	Group I	C-X4-C-X22-H-X1-H	-	-
*MusaWRKY39*	4	GSMUA_Achr4T13970_001	WRKYGQK	Group I	C-X4-C-X22-H-X1-H	-	1
*MusaWRKY40*	4	GSMUA_Achr4T14270_001	WRKYGQK	Group I	C-X4-C-X22-H-X1-H	-	2
*MusaWRKY44*	4	GSMUA_Achr4T16840_001	WRKYGQK	Group I	C-X4-C-X22-H-X1-H	-	-
*MusaWRKY50*	5	GSMUA_Achr5T04150_001	WRKYGQK	Group I	C-X4-C-X22-H-X1-H	-	-
*MusaWRKY70*	6	GSMUA_Achr6T16760_001	WRKYGQK	Group I	C-X4-C-X22-H-X1-H	-	-
*MusaWRKY80*	7	GSMUA_Achr7T00610_001	WRKYGQK	Group I	C-X4-C-X22-H-X1-H	LXXL	-
*MusaWRKY100*	7	GSMUA_Achr7T26450_001	WRKYGQK	Group I	C-X4-C-X22-H-X1-H	-	1
*MusaWRKY130*	10	GSMUA_Achr10T11580_001	WRKYGQK	Group I	C-X4-C-X22-H-X1-H	-	2
*MusaWRKY143*	11	GSMUA_Achr11T10010_001	WRKYGQK	Group I	C-X4-C-X22-H-X1-H	-	-
*MusaWRKY19*	3	GSMUA_Achr3T13440_001	WRKYGQK	Group II-a	C-X5-C-X23-H-X1-H	-	-
*MusaWRKY57*	5	GSMUA_Achr5T16460_001	WRKYGQK	Group II-a	C-X5-C-X23-H-X1-H	-	-
*MusaWRKY81*	7	GSMUA_Achr7T05200_001	WRKYGQK	Group II-a	C-X5-C-X23-H-X1-H	LXLXLXL	-
*MusaWRKY93*	7	GSMUA_Achr7T19340_001	WRKYGQK	Group II-a	C-X5-C-X23-H-X1-H	-	-
*MusaWRKY127*	10	GSMUA_Achr10T06050_001	WRKYGQK	Group II-a	C-X5-C-X23-H-X1-H	LXLXLXL	1
*MusaWRKY136*	10	GSMUA_Achr10T24080_001	WRKYGQK	Group II-a	C-X5-C-X23-H-X1-H	-	-
*MusaWRKY142*	11	GSMUA_Achr11T08290_001	WRKYGQK	Group II-a	C-X5-C-X23-H-X1-H	-	-
*MusaWRKY10*	2	GSMUA_Achr2T15200_001	WRKYGQK	Group II-b	C-X6-C-X29-H-X1-H	LZ	1
*MusaWRKY58*	5	GSMUA_Achr5T16750_001	WRKYGQK	Group II-b	C-X6-C-X29-H-X1-H	-	1
*MusaWRKY62*	6	GSMUA_Achr6T02330_001	WRKYGQK	Group II-b	C-X6-C-X29-H-X1-H	LXLXLXL	1
*MusaWRKY66*	6	GSMUA_Achr6T05880_001	WRKYGQK	Group II-b	C-X6-C-X29-H-X1-H	-	1
*MusaWRKY68*	6	GSMUA_Achr6T07620_001	WRKYGQK	Group II-b	C-X6-C-X29-H-X1-H	LZ, LXLXLXL	-
*MusaWRKY69*	6	GSMUA_Achr6T13630_001	WRKYGQK	Group II-b	C-X6-C-X29-H-X1-H	-	1
*MusaWRKY83*	7	GSMUA_Achr7T08850_001	WRKYGQK	Group II-b	C-X6-C-X29-H-X1-H	-	1
*MusaWRKY92*	7	GSMUA_Achr7T18820_001	WRKYGQK	Group II-b	C-X6-C-X29-H-X1-H	LXLXLXL	-
*MusaWRKY132*	10	GSMUA_Achr10T15290_001	WRKYGQK	Group II-b	C-X6-C-X29-H-X1-H	-	1
*MusaWRKY140*	10	GSMUA_Achr10T30080_001	WRKYGQK	Group II-b	C-X6-C-X29-H-X1-H	LXLXLXL	-
*MusaWRKY145*	11	GSMUA_Achr11T17620_001	WRKYGQK	Group II-b	C-X6-C-X29-H-X1-H	LZ	1
*MusaWRKY1*	1	GSMUA_Achr1T04770_001	WRKYGQK	Group II-c	C-X4-C-X23-H-X1-H	-	1
*MusaWRKY11*	2	GSMUA_Achr2T16390_001	WRKYGQK	Group II-c	C-X4-C-X23-H-X1-H	-	-
*MusaWRKY33*	4	GSMUA_Achr4T02800_001	WRKYGQK	Group II-c	C-X4-C-X23-H-X1-H	-	-
*MusaWRKY41*	4	GSMUA_Achr4T14990_001	WRKYGQK	Group II-c	C-X4-C-X23-H-X1-H	-	1
*MusaWRKY71*	6	GSMUA_Achr6T22160_001	WRKYGQK	Group II-c	C-X4-C-X23-H-X1-H	-	1
*MusaWRKY74*	6	GSMUA_Achr6T27090_001	WRKYGQK	Group II-c	C-X4-C-X23-H-X1-H	-	-
*MusaWRKY85*	7	GSMUA_Achr7T13310_001	WRKYGQK	Group II-c	C-X4-C-X23-H-X1-H	-	-
*MusaWRKY98*	7	GSMUA_Achr7T25400_001	WRKYGQK	Group II-c	C-X4-C-X23-H-X1-H	-	1
*MusaWRKY104*	8	GSMUA_Achr8T10740_001	WRKYGQK	Group II-c	C-X4-C-X23-H-X1-H	-	1
*MusaWRKY112*	9	GSMUA_Achr9T01400_001	WRKYGQK	Group II-c	C-X4-C-X23-H-X1-H	-	-
*MusaWRKY116*	9	GSMUA_Achr9T06750_001	WRK——	Group II-c	C-X4-C-X23-H-X1-H	-	1
*MusaWRKY124*	10	GSMUA_Achr10T00810_001	WRKYGQK	Group II-c	C-X4-C-X23-H-X1-H	-	-
*MusaWRKY138*	10	GSMUA_Achr10T27340_001	WRKYGQK	Group II-c	C-X4-C-X23-H-X1-H	-	2
*MusaWRKY149*	UND	GSMUA_AchrUn_randomT17570_001	WRKYGQK	Group II-c	C-X4-C-X23-H-X1-H	-	1
*MusaWRKY150*	UND	GSMUA_AchrUn_randomT17930_001	WRKYGQK	Group II-c	C-X4-C-X23-H-X1-H	-	1
*MusaWRKY6*	1	GSMUA_Achr1T23560_001	WRKYGQK	Group II-d	C-X5-C-X23-H-X1-H	HARF, LXLXLXL	-
*MusaWRKY15*	3	GSMUA_Achr3T00510_001	WRKYGQK	Group II-d	C-X5-C-X23-H-X1-H	HARF	-
*MusaWRKY17*	3	GSMUA_Achr3T05670_001	WRKYGQK	Group II-d	C-X5-C-X23-H-X1-H	HARF	-
*MusaWRKY25*	3	GSMUA_Achr3T27880_001	WRKYGQK	Group II-d	C-X5-C-X23-H-X1-H	HARF	-
*MusaWRKY26*	3	GSMUA_Achr3T29310_001	WRKYGQK	Group II-d	C-X5-C-X23-H-X1-H	-	-
*MusaWRKY34*	4	GSMUA_Achr4T03660_001	WRKYGQK	Group II-d	C-X5-C-X23-H-X1-H	-	-
*MusaWRKY46*	4	GSMUA_Achr4T20600_001	WRKYGQK	Group II-d	C-X5-C-X23-H-X1-H	HARF	-
*MusaWRKY51*	5	GSMUA_Achr5T04870_001	WRKYGQK	Group II-d	C-X5-C-X23-H-X1-H	LXLXLXL	2
*MusaWRKY60*	5	GSMUA_Achr5T21910_001	WRKYGQK	Group II-d	C-X5-C-X23-H-X1-H	HARF	1
*MusaWRKY90*	7	GSMUA_Achr7T15930_001	WRKYGQK	Group II-d	C-X5-C-X23-H-X1-H	HARF	1
*MusaWRKY108*	8	GSMUA_Achr8T20660_001	WRKYGQK	Group II-d	C-X5-C-X23-H-X1-H	-	-
*MusaWRKY121*	9	GSMUA_Achr9T21560_001	WRKYGQK	Group II-d	C-X5-C-X23-H-X1-H	-	-
*MusaWRKY122*	9	GSMUA_Achr9T22950_001	WRKYGQK	Group II-d	C-X5-C-X23-H-X1-H	HARF	-
*MusaWRKY129*	10	GSMUA_Achr10T08720_001	WRKYGQK	Group II-d	C-X5-C-X23-H-X1-H	LXXL	-
*MusaWRKY135*	10	GSMUA_Achr10T23420_001	WRKYGQK	Group II-d	C-X5-C-X23-H-X1-H	HARF	-
*MusaWRKY20*	3	GSMUA_Achr3T15690_001	WRKYGQK	Group II-e	C-X5-C-X23-H-X1-H	-	-
*MusaWRKY42*	4	GSMUA_Achr4T15540_001	WRKYGQK	Group II-e	C-X5-C-X23-H-X1-H	-	-
*MusaWRKY55*	5	GSMUA_Achr5T09430_001	WRKYGQK	Group II-e	C-X5-C-X23-H-X1-H	-	-
*MusaWRKY75*	6	GSMUA_Achr6T31840_001	WRKYGQK	Group II-e	C-X5-C-X23-H-X1-H	LXXL	2
*MusaWRKY97*	7	GSMUA_Achr7T24840_001	WRKYGQK	Group II-e	C-X5-C-X23-H-X1-H	-	-
*MusaWRKY105*	8	GSMUA_Achr8T14610_001	WRKYGQK	Group II-e	C-X5-C-X23-H-X1-H	-	-
*MusaWRKY107*	8	GSMUA_Achr8T19810_001	WRKYGQK	Group II-e	C-X5-C-X23-H-X1-H	-	-
*MusaWRKY113*	9	GSMUA_Achr9T03020_001	WRKYGQK	Group II-e	C-X5-C-X23-H-X1-H	-	-
*MusaWRKY115*	9	GSMUA_Achr9T05460_001	WRKYGQK	Group II-e	C-X5-C-X23-H-X1-H	-	-
*MusaWRKY125*	10	GSMUA_Achr10T01150_001	WRKYGQK	Group II-e	C-X5-C-X23-H-X1-H	-	-
*MusaWRKY134*	10	GSMUA_Achr10T22020_001	WRKYGQK	Group II-e	C-X5-C-X23-H-X1-H	-	-
*MusaWRKY141*	11	GSMUA_Achr11T06260_001	WRKYGQK	Group II-e	C-X5-C-X23-H-X1-H	-	3
*MusaWRKY146*	11	GSMUA_Achr11T17950_001	WRKYGQK	Group II-e	C-X5-C-X23-H-X1-H	-	1
*MusaWRKY7*	1	GSMUA_Achr1T27980_001	WRKYGQK	Group III	C-X7-C-X23-H-X1-H	LXXL	-
*MusaWRKY24*	3	GSMUA_Achr3T25080_001	WRKYGQK	Group III	C-X7-C-X23-H-X1-H	LXXL	-
*MusaWRKY43*	4	GSMUA_Achr4T15640_001	**WRKYGNK**	Group III	C-X7-C-X23-H-X1-H	LXLXLXL	-
*MusaWRKY65*	6	GSMUA_Achr6T05710_001	WRKYGQK	Group III	C-X7-C-X23-H-X1-H	-	-
*MusaWRKY87*	7	GSMUA_Achr7T14140_001	WRKYGQK	Group III	C-X7-C-X23-H-X1-H	LXXL	-
*MusaWRKY101*	8	GSMUA_Achr8T01730_001	WRKYGQK	Group III	C-X7-C-X23-H-X1-H	-	-

Abbreviations: UND,Undetermined; Chr, Chromosome; CD's, Complete Domains; SSR, Simple Sequence Repeats

The variants of the conserved WRKYGQK peptide are shown in bold.

### Expansion of *MusaWRKY* for resistance

Expansion of transcription factor families particularly in *Musa* is significantly greater than other plant genomes after genome duplication [13]. *Musa* polyploidization events had occurred from selective pressure causing both variation in *cis*-regulatory elements (S6 Table and S2 Fig) and duplication of WRKYs with new activities [28]. Based on the general WRKY functional role, we hypothesized that *WRKY* duplication events may contribute to the fittest survival amidst various biotic and abiotic stresses. First, we looked up for the duplicated paralogous *WRKY* gene clusters corresponding to the 12 synteny blocks developed by D’Hont and colleagues [13, 29] that represent the *Musa* ancestral genome within last three rounds of WGD: α, β and γ (Fig 1). Synteny blocks are homologous regions in chromosomes with a series of genes residing in same order corresponding to a common ancestral genomic region. Using synteny analyses data (S7 Table), paralogous relationships were derived based on the synonymous substitution rate (Ks), which is a function of genomic evolutionary events. Ks distribution among pairs of paralogous gene clusters indicates the α+ß WGD events (Ks ~ 0 to 0.45) and the γ WGD event (Ks ~ 0.45 to 0.85) that occurred 64.8MYA and 96MYA respectively in *Musa* lineage [13]. Two paralog gene pairs, *MusaWRKY40*/*MusaWRKY100* and *MusaWRKY50*/*MusaWRKY143*, were identified from the α+ß WGD events. Fifteen paralogous WRKY gene pairs were identified from the γ WGD event: *MusaWRKY04*/*MusaWRKY08*, *MusaWRKY20*/*MusaWRKY111*, *MusaWRKY26*/*MusaWRKY121*,*MusaWRKY27*/*MusaWRKY39*, *MusaWRKY42*/*MusaWRKY97*, *MusaWRKY62*/*MusaWRKY92*, *MusaWRKY62*/*MusaWRKY140*,*MusaWRKY66*/*MusaWRKY137*, *MusaWRKY70*/*MusaWRKY130*, *MusaWRKY74*/*MusaWRKY116*, *MusaWRKY87*/*MusaWRKY133*, *MusaWRKY105*/*MusaWRKY146*,*MusaWRKY107*/*MusaWRKY134*,*MusaWRKY118*/*MusaWRKY108* and *MusaWRKY118*/*MusaWRKY129*. Among the 15 collinear relationships, only *MusaWRKY62* and *MusaWRKY118* have multiple paralogs (S7 Table and Fig 1). All duplicated genes were collinear with ancestral blocks and existed across chromosomes, suggesting that segmental duplication had significantly contributed to the expansion of WRKY gene family in *Musa* as similar to rice. Separately, we identified 15 *MusaWRKY* gene paralogs (Ks>0.85) attributing probably to older duplication events that may be inferred to have occurred along Arecales and Poales lineage of commelinids (S7 Table). Additionally, by Holub method [30] eight *MusaWRKY* genes could be placed in four tandem clusters (Fig 1 and S1 Fig), two on chromosome seven and one on chromosomes eight and ten. On chromosome seven, *MusaWRKY79* and *-80* are clustered together in opposite orientation to each other on a 30-kb fragment and similarly *MusaWRKY96* and *-97* are clustered in opposite orientation on a 37-kb fragment. *MusaWRKY101* and -*102* are clustered together in opposite orientation on a 36-kb fragment of chromosome eight whereas *MusaWRKY127* and -*128* are clustered together in the same orientation on a 7-kb fragment of chromosome ten (S1 Fig). Among the four tandem clusters, only three gene pairs were found to be tandemly duplicated: *MusaWRKY79*/*MusaWRKY80*, *MusaWRKY96*/*MusaWRKY97* and *MusaWRKY101*/*MusaWRKY102*. Further by COGE analysis, *MusaWRKY85*/*MusaWRKY86*was also identified to be from tandem duplication. Thus, in *Musa* tandem duplication events are much lesser than the segmental duplication as similar to rice [31]. In this transcriptome study, expression data were available only for four segmentally duplicated *MusaWRKY* pairs and the expression pattern of both copies of all paralogous *MusaWRKY* pairs was similar (either both upregulated or down regulated). Noticeably, there was expression divergence among one tandemly duplicated gene pair, *MusaWRKY85/86*, in which *MusaWRKY85* was getting downregulated in contrast to its duplicated copy, *MusaWRKY86*, which was upregulated in susceptible cultivar after nematode challenge. We then predicted the divergence functions of duplicated genes from Ω ratio, the ratio of the number of nonsynonymous substitutions per nonsynonymous site (Ka) to the number of synonymous substitutions per synonymous site (Ks) [32]. The Ω ratio (Ka/Ks) for all the paralog *WRKY* gene pairs was found to be less than one suggesting the fate of duplicated WRKY genes as sub-functionalization [33] (S7 Table) in which the duplicated gene copies continue maintaining a subset of theancestral gene function. Thus, *WRKY* gene copies were undergoing purifying selections in the *Musa* evolution thereby contributing to phenotypic variation in polyploids. Such evolutionary changes had a synergistic impact on WRKYs in acquiring new binding specificities thereby governing various signaling mechanism with respect to defense: JA, SA, ISR (Induced Systemic Resistance), SAR (Systemic Acquired Resistance) and PR genes (Pathogenesis-Related) like peroxidase and catalase [4, 5, 6, 7, 14].

### Expression patterns of *MusaWRKYs* in compatible and incompatible interactions of *Musa-P*.*coffeae*

Several WRKYs have been directly associated with defense against nematodes such as rice OsWRKY59, -62, -70 and -11, tomato SlWRKY70 and *Arabidopsis* AtWRKY35, 45 and 48 (S8 Table) [15, 34–39]. To find MusaWRKYs that are involved in nematode stress response, the differential gene expressions of *MusaWRKY*s were shortlisted from the cuffdiff analysis of RNAseq data on nematode resistant and susceptible cultivars during nematode challenged and unchallenged conditions (Table 2). Altogether, during challenged and unchallenged conditions among 153 *MusaWRKY*s identified from *Musa* genome, 99*MusaWRKY*s were expressed in resistant cultivar (S9 Table) and only 91*MusaWRKY*s were expressed in susceptible cultivar (S10 Table). The non-detection of remaining *MusaWRKY*s in control and nematode challenged root tissues of resistant and susceptible cultivars may be due to insufficient depth and coverage or that these *MusaWRKYs* may not be associated with root development and/or nematode stress response. During unchallenged conditions, 93 and 79*MusaWRKYs* were found to be expressed in resistant and susceptible cultivars respectively (S9 and S10 Tables) and this constitutive expression indicates their functional role in root development.

**Table 2:** Differential regulation of *MusaWRKY*s in resistant and susceptible cultivars during *P*.*coffeae* infestation.

		Resistant cultivar		Susceptible cultivar	
*Musa* genome hub ID	*MusaWRKY* name	Log2 fold change	*P*-value	Log2 fold change	*P*-value
GSMUA_Achr1G05010_001	*MusaWRKY2*	5.15	2.02e-07	-	-
GSMUA_Achr3G09940_001	*MusaWRKY18*	2.28	0.00	2.41	0.004
GSMUA_Achr9G19580_001	*MusaWRKY120*	1.37	0.05	-	-
GSMUA_Achr10G11580_001	*MusaWRKY130*	-	-	1.82	0.02
GSMUA_Achr1G17160_001	*MusaWRKY5*	-	-	1.71	0.01
GSMUA_Achr10G06050_001	*MusaWRKY127*	1.56	0.01	-	-
GSMUA_Achr10G24080_001	*MusaWRKY136*	Inf	0.04	-	-
GSMUA_Achr11G08290_001	*MusaWRKY142*	4.18	1.50e-07	2.93	0.00
GSMUA_Achr3G13440_001	*MusaWRKY19*	4.15	1.76e-07	4.1	2.75e-06
GSMUA_Achr4G01930_001	*MusaWRKY32*	2.85	0.05	Inf	0.00
GSMUA_Achr5G16460_001	*MusaWRKY57*	Inf	0.03	-	-
GSMUA_Achr7G05200_001	*MusaWRKY81*	4.46	9.59e-09	2.22	0.00
GSMUA_Achr7G16090_001	*MusaWRKY91*	6.93	1.25e-08	-	-
GSMUA_Achr7G19340_001	*MusaWRKY93*	3.37	0.00	3.09	0.00
GSMUA_Achr2G05100_001	*MusaWRKY8*	-	-	Inf	0.02
GSMUA_Achr11G17620_001	*MusaWRKY145*	3.24	0.00	-	-
GSMUA_Achr2G18390_001	*MusaWRKY13*	3.06	0.00	-	-
GSMUA_Achr6G13630_001	*MusaWRKY69*	-2.16	0.01	4.65	0.00
GSMUA_Achr7G18820_001	*MusaWRKY92*	-2.22	0.00	Inf	0.02
GSMUA_Achr10G15290_001	*MusaWRKY132*	-	-	1.78	0.00
GSMUA_Achr10G26050_001	*MusaWRKY137*	-	-	2.11	0.00
GSMUA_Achr3G03090_001	*MusaWRKY16*	-	-	Inf	0.02
GSMUA_Achr3G20290_001	*MusaWRKY22*	-	-	Inf	0.01
GSMUA_Achr7G13400_001	*MusaWRKY86*	-	-	Inf	0.02
GSMUA_Achr3G20450_001	*MusaWRKY23*	5.77	3.70e-05	4.61	0.00
GSMUA_Achr3G31310_001	*MusaWRKY30*	4.19	1.81e-07	3.14	3.15e-05
GSMUA_Achr4G02800_001	*MusaWRKY33*	Inf	0.02	Inf	0.00
GSMUA_Achr4G07920_001	*MusaWRKY36*	2.99	0.01	2.44	0.01
GSMUA_Achr4G08880_001	*MusaWRKY38*	5.19	4.26e-05	6.18	2.60e-05
GSMUA_Achr4G14990_001	*MusaWRKY41*	2.17	0.00	1.92	0.00
GSMUA_Achr4G23360_001	*MusaWRKY47*	3.19	0.00	-	-
GSMUA_Achr5G06260_001	*MusaWRKY52*	2.26	0.02	-1.81	0.03
GSMUA_Achr6G22160_001	*MusaWRKY71*	2.89	0.00	Inf	0.00
GSMUA_Achr6G22920_001	*MusaWRKY72*	4.84	5.22e-07	-	-
GSMUA_Achr6G27090_001	*MusaWRKY74*	4.31	7.56e-08	5.66	1.62e-10
GSMUA_Achr7G00580_001	*MusaWRKY79*	Inf	0.05	-	-
GSMUA_Achr7G06860_001	*MusaWRKY82*	3.37	0.02	-	-
GSMUA_Achr7G09680_001	*MusaWRKY84*	2.71	0.00	2.76	0.00
GSMUA_Achr7G23980_001	*MusaWRKY95*	5.10	5.85e-05	-	-
GSMUA_Achr7G24780_001	*MusaWRKY96*	Inf	0.02	-	-
GSMUA_Achr7G25400_001	*MusaWRKY98*	2.43	0.00	-	-
GSMUA_Achr8G02180_001	*MusaWRKY103*	2.46	0.01	-	-
GSMUA_Achr8G26240_001	*MusaWRKY110*	2.00	0.02	2.52	0.01
GSMUA_Achr9G01400_001	*MusaWRKY112*	6.63	4.00e-11	2.11	0.00
GSMUA_Achr9G07230_001	*MusaWRKY117*	3.99	1.46e-05	-	-
GSMUA_Achr9G29350_001	*MusaWRKY123*	Inf	0.02	Inf	0.01
GSMUA_Achr1G04770_001	*MusaWRKY1*	-	-	Inf	0.01
GSMUA_Achr2G16390_001	*MusaWRKY11*	-	-	2.28	0.01
GSMUA_Achr6G06360_001	*MusaWRKY67*	-	-	7.03	0.00
GSMUA_Achr7G13310_001	*MusaWRKY85*	-	-	-1.72	0.01
GSMUA_Achr7G15060_001	*MusaWRKY89*	-	-	2.04	0.03
GSMUA_Achr9G06750_001	*MusaWRKY116*	-	-	2.02	0.02
GSMUA_Achr10G08720_001	*MusaWRKY129*	1.57	0.01	-	-
GSMUA_Achr10G23420_001	*MusaWRKY135*	1.66	0.03	-	-
GSMUA_Achr6G03810_001	*MusaWRKY64*	Inf	0.05	- Inf	0.04
GSMUA_Achr10G14710_001	*MusaWRKY131*	Inf	0.03	-	-
GSMUA_Achr4G27420_001	*MusaWRKY48*	-2.07	0.03	-	-
GSMUA_Achr7G14300_001	*MusaWRKY88*	2.04	0.05	-	-
GSMUA_Achr1G27980_001	*MusaWRKY7*	1.93	0.00	-	-
GSMUA_Achr4G15640_001	*MusaWRKY43*	Inf	0.04	-	-
GSMUA_Achr5G07490_001	*MusaWRKY53*	2.09	0.00	-	-
GSMUA_Achr7G14140_001	*MusaWRKY87*	-	-	1.91	0.00

**Fig 4:**
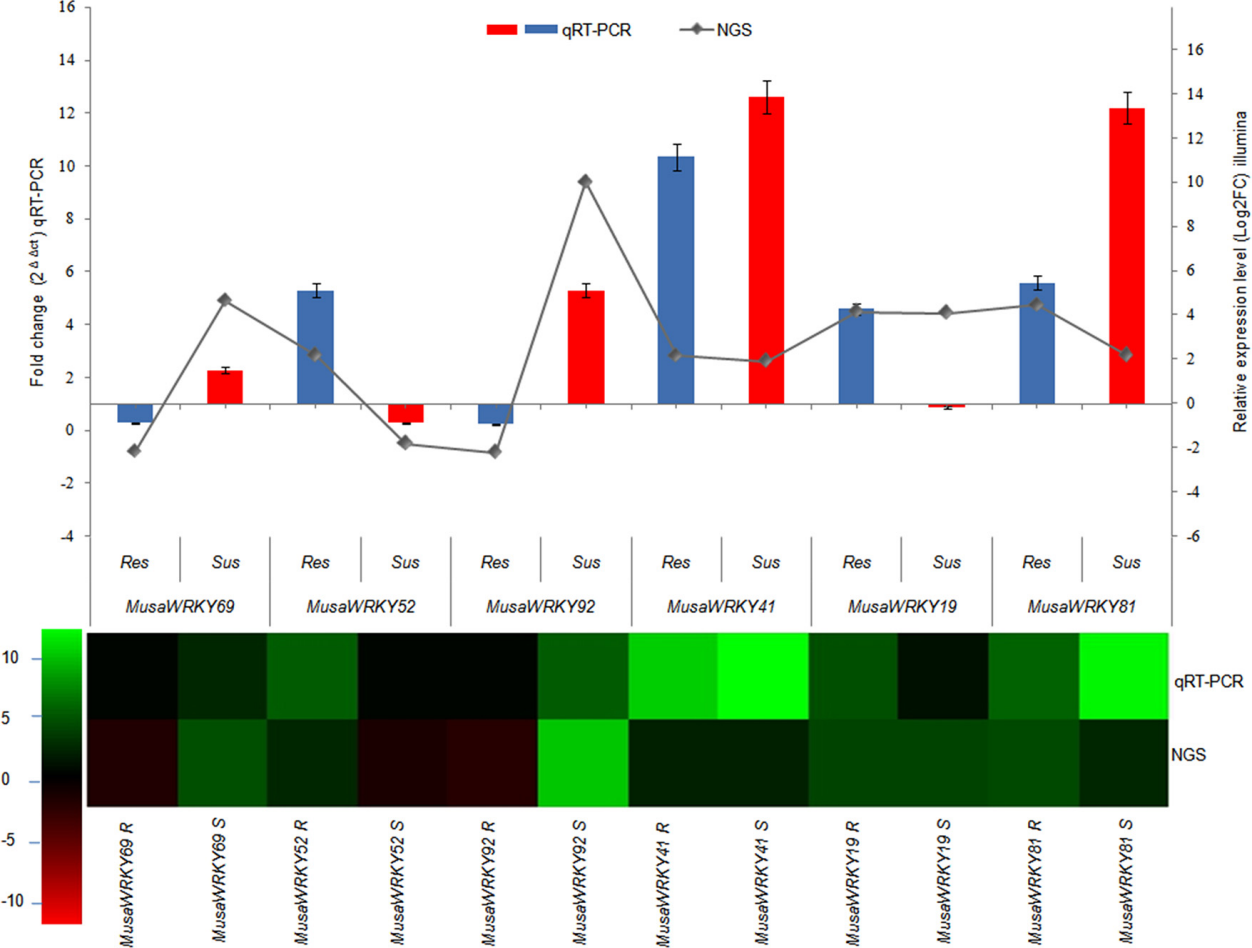
Quantitative RT-PCR validation of DGE analysis. Relative quantification was carried out to measure changes of selective WRKY gene expression in *P*.*coffeae* resistant and susceptible root sample relative to an endogenous reference gene (RPS2). Red line shows the expression analysis in DGE. Data (technical triplicates of three biological experiments) are reported as means ± standard error.

Upon infection, 49 *MusaWRKY*s were found to be differentially regulated in resistant cultivar and 40*MusaWRKY*s were differentially regulated in susceptible cultivar significantly with majority of them from group IIc (42 *MusaWRKY*s). Among them, upregulation of 20 *MusaWRKY*s was common to both susceptible and resistant cultivars, indicating their participation in general banana response mechanism against *P*.*coffeae*.23 *MusaWRKY*s showed significantly increased transcript levels that were unique to the resistant line in *P*.*coffeae* infected root tissue as compared to control, indicating their role in resistance mechanism and 15 *MusaWRKY*s were uniquely upregulated only in susceptible cultivar pointing that some of these *MusaWRKY*s may aid in the pathogen survival as suppressors of plant triggered immunity. *MusaWRKY*s 31, -48 and -131 were uniquely downregulated only in resistant cultivar where as *MusaWRKY*85 was downregulated in susceptible cultivar. Interestingly, three *MusaWRKY*s,-52, -69 and -92 showed a contrasting regulation among the susceptible and resistant cultivar and hence can be considered as cultivar specific response. Mainly, *MusaWRKY*52 was twofold higher in resistant cultivar while getting significantly downregulated in susceptible cultivar, suggesting that *MusaWRKY52* may be a *P*.*coffeae* specific response; *MusaWRKY*69 and *MusaWRKY*92 were highly upregulated in susceptible cultivar but downregulated in resistant cultivar speculating that it may be acting as a repressor in a defense pathway. To validate the differential expression patterns of DEGs, six functional genes were selected for RT-qPCR analysis: *MusaWRKY52*, *MusaWRKY69*, *MusaWRKY92*, *MusaWRKY41*, *MusaWRKY19* and *MusaWRKY81*. Their expression trends were similar to the transcript abundance changes by RNA-Seq indicating the quality of illumina sequencing (Fig 4).

## Conclusion

WRKY transcription factors play multiple roles in plant growth and development, production of secondary metabolites and defense pathways. A large evolutionary expansion of this master transcriptional regulator in *Musa* may be a strategy for better performance of these plants to survive against biotic and abiotic stresses. After nematode attack, there was a significant change in expression patterns of many *MusaWRKY*s indicating their participation in the nematode stress response. In particular, *MusaWRKY52*, *-69* and *-92* showed a contrasting regulation between resistant and susceptible cultivar during nematode attack. Due to the limited availability of *MusaWRKY* studies, an in-depth study on the differentially regulated *WRKY*s is further needed to understand the actual *WRKY* gene regulatory networks during *Musa*- *P*.*coffeae* interaction. Our study based on the computational analysis and differential gene expression analysis of *MusaWRKY* gene family provide a foundation for further detailed studies on *MusaWRKY*.

## Supporting Information

S1 Fig Tandem clusters of MusaWRKY79, -80, -96 and -97 (chr 7), -101, -102 (chr 8), -127, -128 (chr 10) from single ancestral genes on chromosomes. This is shown in the screen shot from BGH database G-browser, the position and orientation indicated by arrows.
(TIF)

S2 Fig Various cis-acting elements in MusaWRKY’s and Orthologs of rice WRKY’s. (TIF)

S1 Table Information about the illumina sequencing data. (DOCX)

S2 Table Quantitative RT-PCR primer sequences of MusaWRKYs. (XLSX)

S3 Table Chromosomal location of WRKY genes in Musa. (XLSX)

S4 Table List of MusaWRKYs having LXXL, Leucine zipper, HARF and LXLXL motifs. (XLSX)

S5 Table Physicochemical properties of Complete CD's of MusaWRKY's. (XLSX)

S6 Table Comparison of commonly available cis-regulatory elements among MusaWRKYs with rice WRKYs orthologs. (XLSX)

S7 Table Evolutionary expansion of MusaWRKY genes through duplicated events evidenced by synonymous and non synonymous substitution rate analysis. (XLSX)

S8 Table Expression of WRKY gene family in various plant species against nematodes. (XLSX)

S9 Table Differential Gene Expression (DGE) of MusaWRKYs in nematode resistant cultivar. (XLSX)

S10 Table Differential Gene Expression (DGE) of MusaWRKYs in nematode susceptible cultivar. (XLSX)

S11 Table Motif analysis in MusaWRKYs. (XLSX)
