# Supplementary figures and images for: Evolutionary Expansion of WRKY Gene Family in Banana and Its Expression Profile during the Infection of Root Lesion Nematode, Pratylenchus coffeae
Source: PLoS One. 2016 Sep 7;11(9):e0162013. doi: 10.1371/journal.pone.0162013 (PMC5014340; doi:10.1371/journal.pone.0162013)

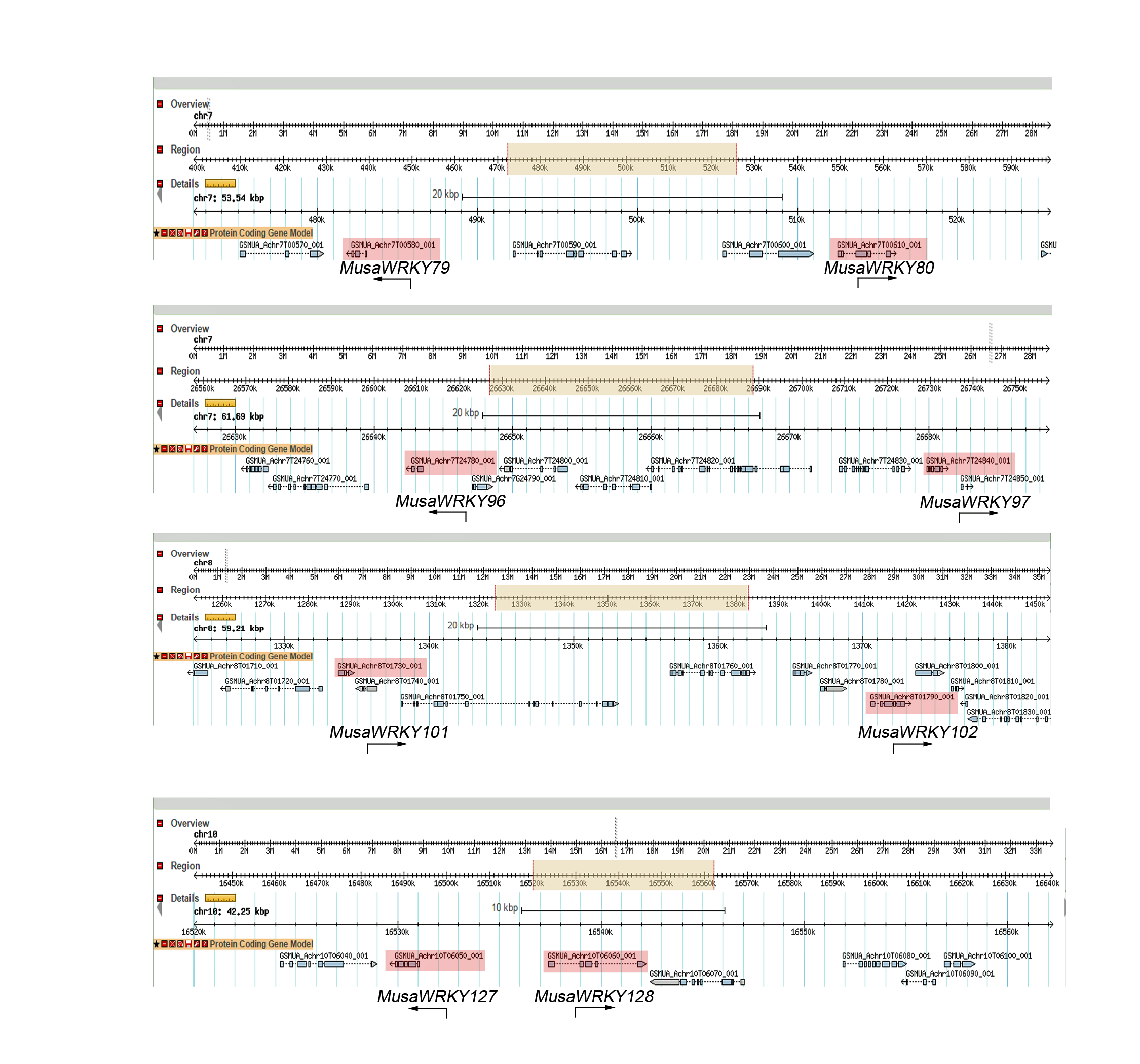

Supplement: S1 Fig — This is shown in the screen shot from BGH database G-browser, the position and orientation indicated by arrows. (TIF) [file pone.0162013.s001.tif]

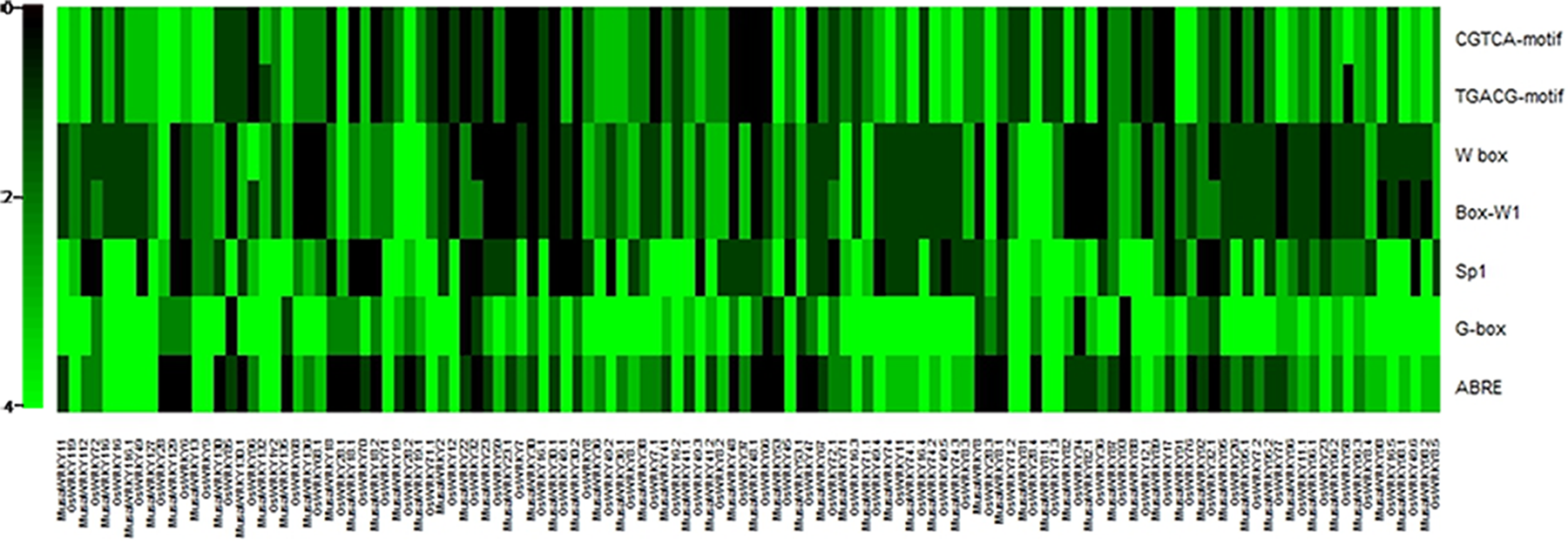

Supplement: S2 Fig — (TIF) [file pone.0162013.s002.tif]
