# Supplementary material for: Evolutionary Expansion of WRKY Gene Family in Banana and Its Expression Profile during the Infection of Root Lesion Nematode, Pratylenchus coffeae
Source: PLoS One. 2016 Sep 7;11(9):e0162013. doi: 10.1371/journal.pone.0162013 (PMC5014340; doi:10.1371/journal.pone.0162013)
Supplement: S1 Table — (DOCX) [file pone.0162013.s003.docx]

Supplementary file 1. Information about the illumina sequencing data.

| **Parameters/Libraries** | **UR** | **CR** | **US** | **CS** |
| --- | --- | --- | --- | --- |
| Total base pairs (bp) | 3554530092 | 2635191600 | 3210859189 | 3475216904 |
| Total number of reads | 35909656 | 26351916 | 32415970 | 35075840 |
| Percentage of HQ reads | 97.914% | 97.91% | 97.54% | 100.00% |
| GC percentage | 48.94% | 45.99% | 47.82% | 47.35% |
| Total number of contigs | 54556 | 48936 | 54338 | 47205 |
| Mean length of contigs (bp) | 1184 | 847.7 | 951.3 | 954.3 |
| Maximum contig length | 15392 | 11298 | 15700 | 11588 |
| Minimum contig length | 63 | 70 | 70 | 66 |
